# An Insight into developmental changes in reasoning skills among Indian Preschoolers: A cross-sectional study using a story-based approach

**DOI:** 10.12688/f1000research.131906.1

**Published:** 2023-04-26

**Authors:** Aparna Prasanna, Gagan Bajaj, Malavika Anakkathil Anil, Jayashree S Bhat

**Affiliations:** 1 Department of Audiology and Speech-Language Pathology, Kasturba Medical College, Mangalore, Manipal Academy of Higher Education, Manipal, Karnataka, 575001, India; 2 The MARCS Institute for Brain, Behaviour, and Development, Western Sydney University, Sydney, 2145, Australia; 3 Nitte Institute of Speech and Hearing, Mangalore, Karnataka, 575018, India

**Keywords:** explanation, prediction, inference, reasoning, development, story, preschoolers, India

## Abstract

**Background:** Considering the importance of exploring the development of reasoning skills during preschool period and the suitability of using a culturally linguistically relevant story-based approach for the same, the present research intended to profile the reasoning skills in typically developing Indian preschool children between 36 and 72 months using a story-based approach. The specific objectives were to develop explanation, prediction, and inference based reasoning tasks around a story and assess the reasoning skills in typically developing Indian preschool children using the same.

**Method:** Reasoning tasks across explanation, prediction, and inference domains were developed based on a story and evaluated for its psychometric properties. The developed tasks were then administered to 63 typically developing Indian preschool children attending English medium schools in Mangalore. The preschoolers were equally divided into three age groups, and the responses obtained across the age groups were analyzed quantitatively and qualitatively.

**Results:** The developed tasks were confirmed to have good psychometric properties like content validity and reliability. The age comparisons of reasoning abilities using one-way ANOVA suggested an increase in reasoning abilities with age during the preschool period. The qualitative analysis further suggested that with increasing age, the nature of reasoning changed from content-based reasoning to reasoning based on prior knowledge which was integrated with the story content.

**Conclusion:** The study describes reasoning skill development using a story-based task in Indian preschoolers. The study findings further provide clinical and educational implications to assess and foster reasoning abilities among preschoolers.

## Introduction

Reasoning is a cognitive process of making inferences, drawing conclusions, or evaluating a proposed conclusion ( Andrews, 2020). Reasoning skills enable individuals to understand and learn about the physical and social environment on a daily basis ( Khemlani, 2018). The ability to appropriately reason across different life situations is essential for a successful professional and everyday life ( Bronkhorst *et al.*, 2020). It was long believed that reasoning skills do not develop until adolescence ( Piaget, 1952). Although preschoolers are known as ‘little scientists’ with abundant curiosity and an innate drive to know the world ( Alvarez & Booth, 2014; Piaget, 1952), reasoning skills are considered to be a higher-level cognitive skill that is beyond the reach of preschoolers ( Whittaker & McMullen, 2014). Thinking abilities among preschoolers have often been described as “egocentric, pre-logical, affective, undifferentiated, pre-causal, personal, vague and unanalysed” ( Piaget, 1952). However, some recent researchers indicate that the development of reasoning skills begins during the preschool years and continues through adolescence ( Amsterlaw, 2006; Gopnik *et al.*, 2004; Legare *et al.*, 2010; Säre, Luik & Fisher, 2016a; Whittaker & McMullen, 2014).

It is essential to explore the development of reasoning skills during the preschool age, as it significantly influences the development of other critical skills like language and literacy ( Bauer & Booth, 2019; Nussipzhanova *et al.*, 2018). Understanding reasoning skills during preschool years is vital for several critical decisions, such as the timely preparation of children to perceive and understand real-life scenarios, the right time for school entry, and laying the foundation for academic achievement and success ( Niklas *et al.*, 2018; Pasnak *et al.*, 2015).

The existing literature suggests that reasoning abilities among preschoolers have been explored in domains like reasoning associated with improbable and less logical events like growing money on a tree, going back in time ( Shtulman & Carey, 2007), counterfactual events ( Muller *et al.*, 2007), judgments on human abilities ( Heyman *et al.*, 2003), temporal ordering of events ( McCormack & Hoerl, 2005), teleological functions ( Kelemen *et al.*, 2003), consistent and inconsistent events ( Legare *et al.*, 2010), pre-energy reasoning ( Koliopoulos *et al.*, 2009), analogies ( Simms *et al.*, 2018), and causal reasoning concerning scientific literacy associated with space, structures, tools, etc. ( Bauer & Booth, 2019). However, the findings from these studies remain inconclusive in understanding the general reasoning development in preschoolers that could be fostered through education during the preschool period ( Säre, Luik, & Fisher, 2016a). These studies seem to focus less on how children verbally reason in a general living context and how education could be the mediator to foster reasoning among preschoolers in such contexts. We could find one such recent initiative by Säre, Luik, and Fisher (2016a), wherein a valid and reliable tool ‘Younger Children Verbal Reasoning Test’ was developed to aid educators in assessing the general verbal reasoning of older preschoolers between five and six years of age. The tool utilized scenarios related to a theme on ‘bravery’ with illustrations and prompted children to judge how brave the characters are in the given scenario based on their actions. However, the authors of the work highlighted the psychometric properties of the developed tool and provided limited description regarding application of their tool in understanding general reasoning skills development in preschoolers.

The stimuli used to assess the reasoning abilities among preschoolers is a significant aspect to consider while discussing the development of general reasoning skills in this age group. In this context, Kendeou *et al.* (2019) put forth an integrated theoretical framework, inferential Language Comprehension (iLC), guiding and recommending the effective use of static or dynamic visual narratives (Stories) as a stimulus for evaluating reasoning skills among younger children. The study recommended that reasoning skills, such as inferencing opportunities, could be increased using static or dynamic visual narrative with questioning techniques in younger children who are non-readers. The story-based materials are reported to aid in better representation of general reasoning abilities in everyday communication contexts as they are more relatable to the natural learning context for the children ( Killick & Boffey, 2012; O’Reilly *et al.*, 2022). Stories also seem to provide an opportunity for children to provide explanations, form predictions, and make inferences that would help them to form logical, causally sequenced plots ( Paris & Paris, 2003). Since a long, stories are known to constitute an inevitable part of childhood education, as children are intrinsically drawn to stories, and stories facilitate the development of thinking and learning in children ( Paley, 2013). Recent research by Dawes *et al.* (2019) advocates the potential and sensitivity of a story-based approach to obtain insight into preschoolers’ developmental changes for skills such as inferential comprehension. The application of story-based stimuli to assess reasoning skills during preschool years has ranged from asking questions in an open-ended format after a short story sequence with picture cards ( Muller *et al.*, 2007; Shtulman & Carey, 2007) or after short fragments of story scenarios depicted through illustrations and objects ( Bauer & Booth, 2019; Heyman *et al.*, 2003; McCormack & Hoerl, 2005; Simms *et al.*, 2018). A recent review by O’Reilly *et al.* (2022) highlighted the effectiveness of stories in fostering critical thinking, such as reasoning and problem-solving among preschoolers, and advocated the utility of story-based approaches in the field of critical thinking for future researchers.

Cognitive assessments, in general, have been recommended to be culturally and linguistically relevant to the age and community of the participants for yielding accurate findings ( Tanveer *et al.*, 2022). Likewise, though stories offer significant potential in assessing reasoning abilities among preschool age children, it is important to note that stories should be culturally and linguistically relevant for getting a true insight into the developmental trends of reasoning abilities among preschoolers ( Rao *et al.*, 2021; Sternberg, 2004).

Some other crucial aspects while exploring general reasoning skills among preschoolers, besides the stimuli, are the ways reasoning based responses are elicited with the children and the different tasks employed to assess their abilities. An interventional research on facilitating reasoning among preschoolers used undirected, shared picture book narration within a peer-group setting ( Reed *et al.*, 2015). The study qualitatively analyzed the narration discourse by preschoolers while they freely generated stories from picture books and observed the emergence of reasoning categories such as explanation, prediction, and inference. The findings of this research informed that spontaneously elicited reasoning categories in pre-schoolers without guidance could better represent the reasoning developmental changes in the preschool period *.* Regarding the tasks for assessing reasoning among preschoolers, different studies have employed tasks based on explanation, predictions, and inferencing. In the explanation-based reasoning tasks, participants are expected to explain the reason behind an outcome ( Legare *et al.*, 2010) whereas, in the prediction-based reasoning tasks, participants are required to predict the outcome of an event assuming a cause ( Bonawitz *et al.*, 2010). The inference-based reasoning task expects the participants to form a conclusion about a connection between an antecedent and an outcome with rational justification ( Gopnik *et al.*, 2004). Similar reasoning tasks based on explanation, prediction, and inference were utilized in preschoolers to explore reasoning skills related to the physical causality of objects by Blank *et al.* (1981). It seems most promising to incorporate explanation, prediction, and inference based reasoning tasks in a story-based approach while studying reasoning development during preschool years.

While realizing the need to explore the developmental trends of reasoning among preschoolers and the potential of culturally-linguistically relevant story-based approach for the same, the present research aimed at profiling the reasoning skills in typically developing Indian preschool children between 36 and 72 months using a story-based approach. The specific objectives of the research were to develop explanation, prediction, and inference based reasoning tasks around a story and assess reasoning skills in typically developing Indian preschool children using the same. The study did not expect the developmental trends of the reasoning abilities to be gender specific based on the evidence from previous literature ( Ardila *et al*., 2011).

## Methods

The study followed a cross-sectional research design following STROBE guidelines and commenced after obtaining Institutional Ethical Clearance from Kasturba Medical College, Mangalore, Manipal Academy of Higher Education (IEC KMC MLR 02-19/51). The data collection was conducted between June 2021 to January 2022 in Mangalore, India.

### Participants

Sixty-three typically developing children aged 36 to 72 months attending English medium schools in Mangalore were selected as participants. Written informed consent was obtained from school authorities, and parents of all the participants before their inclusion in the study. The age and gender of the participants was confirmed based on parental report and the participants were equally divided into three groups according to age, i.e., Group I (Age range: 36–48 months, Mean age: 43 months, Standard deviation: 3.8 months, 10 Females and 11 Males), Group II (Age range: 49–60 months, Mean age: 54 months, Standard deviation: 3.8 months, 10 Females and 11 Males) and Group III (Age range: 61–72 months, Mean age: 65 months, Standard deviation: 3.2 months, 10 Females and 11 Males). Initially, 76 children were screened for typical sensorimotor, cognitive, and language development using the Ten Questions Screen (TQS) ( Durkin *et al.*, 1995) and Assessment of Language Development (ALD) ( Lakkanna *et al.*, 2021). Children who qualified across all the 10 items of the TQS and those who exhibited age-adequate receptive and expressive language skills according to ALD were recruited. Since socioeconomic status highly correlates with the cognitive development of young children ( Greenfield & Moorman, 2019), only those participants who belonged to middle socioeconomic status according to the modified Kuppuswamy socioeconomic scale ( Saleem, 2019) were included in this study. Only those children who had attended nursery before joining preschool were included in the study to ensure homogeneity concerning exposure to literacy skills. Likewise, 13 children were excluded from participation due to either history or complaint of deficits in sensory, motor, and/or language development. All the shortlisted children had English as their second language. Children with English language proficiency of seven or greater on a 10-point rating scale of the Child Language Experience and Proficiency Questionnaire (LEAP-Q-Child version) were included in the study ( Marian *et al.*, 2007; Rochanavibhata *et al.*, 2019).

### Material

A story-based assessment task for reasoning was developed based on the iLC framework proposed by Kendeou *et al.* (2019) for assessing the reasoning skills among preschoolers. This framework emphasized the utility of a static visual narrative and questioning technique for assessing reasoning abilities among preschoolers. Present research used a modified version of a story constructed in our previous work ( Prasanna *et al.*, 2022), wherein the effects of modality on a story-based recall task were assessed among the preschoolers. The story titled ‘A Day at Grandparents House’ ( Prasanna *et al.*, 2021b) with its six pictures ( Prasanna *et al.*, 2021a) was modified concerning its length and distribution of story elements across the modalities of presentation. The modified story, which did not differ with respect to the overall theme of the basic story, comprised three-story sections with two corresponding pictures under each section and 14 story elements per section delivered only through the auditory and auditory-visual modality. The researchers jointly validated the modified story to ensure that the story’s overall theme and linguistic elements, relevant to the preschool age, were retained. The story was designed in English to maintain uniformity among participants from different native languages.

To assess the reasoning abilities, explanation, prediction, and inference based reasoning tasks were formulated for each of the sections of the story. Considering the potential of questioning as a strategy to assess reasoning among young children ( Kendeou *et al.*, 2019; Säre, Luik, & Tulviste, 2016b), the tasks in the present research were designed in a question based format. The questions regarding explanation and prediction based reasoning abilities were framed in an open-ended format (Eg: Why did Virat get scared? [explanation], What would have happened if grandpa had not locked the dog in the cage? [prediction]), whereas the questions for the inference based reasoning comprised of a closed-ended polar question (Yes/No type) to elicit the inference followed by an open-ended question to prompt the justification for the same (Eg: Did Virat love grandparents? [inference] followed by What made you feel that way? [justification for the inference]).

The initial version of the tasks that comprised six explanation, six prediction, and nine inference questions were content validated for their relevance, demand posed on the child, linguistic appropriateness of the instructions, questions, and scoring, using a five-point Likert scale (1 - Highly inappropriate/Highly incomprehensible/Highly irrelevant, 2 - “Inappropriate/Incomprehensive/Irrelevant,” 3 - “Not sure,” 4 - Appropriate/Comprehensive/Relevant, and 5 - “Highly Appropriate/Highly comprehensive/Highly relevant”). Three Speech-Language Pathologists with more than five years of expertise in the field of cognitive communication and three preschool teachers with more than five years of teaching experience with preschoolers performed the content validation. The items with a content validity index greater than 0.78 were included in the assessment protocol ( Almanasreh *et al.*, 2019). Based on this criteria, three questions from the inference based reasoning task were excluded. Further, the feasibility of these tasks was ascertained through a pilot study among 15 typically developing children aged between 36 to 72 months. Since diverse answers could be possible for some of these reasoning tasks, especially among preschoolers, all the probable responses obtained during the pilot study were reviewed and discussed by the researchers to formulate an answer key. To obtain the test-retest reliability, the tasks were re-administered after two weeks among the 15 preschoolers (five from each age group). Since the present study is a preliminary step in tool development, which is planned to be commercially released in the future, the optimum representation of the story text and reasoning tasks has been provided in Table 2 of the Results section for the readers.

### Procedure

The data collection was carried out within the home premises, wherein the participants were seated in a well-lit, quiet room with only the researcher and the child present during the assessment. The evaluation was scheduled in the morning, considering the children’s active time according to the mother’s feedback. The researcher initially built rapport with the children and developed their interest in listening to the story through engaging in an active conversation about stories (For example: Do you like to listen to a story? Does your mother tell you stories? I am going to tell you a story. Would you like to know what happened in that story?). The researcher then instructed the child to carefully listen to the story and answer the questions, which was rewarded with reinforcement (stickers). The researcher also explained that if the child did not know the answer or wanted to think longer, they could freely say that. A practice trial was given to the child by listening to another short story (different from the story used in present assessment) and answering three reasoning-based questions based on the story to familiarise with the assessment procedure.

After the practice trial, the story was presented using a laptop and headphones. The reasoning tasks were performed after the presentation of each story section to ensure that excessive recall load did not dilute the findings. The order of the presentation of the questions in each section was randomized for each participant using a random number generator. Children were encouraged to respond verbally to all the questions and were rewarded with reinforcement (stickers). The assessment took around 20 minutes per participant, including a one-minute rest after each story section and task.

### Data processing and analysis

The psychometric properties of the developed story-based reasoning tasks were evaluated using content validity and test-retest reliability measures. The Content Validity measure at the Item level (I-CVI) was computed by obtaining the ratio of experts who rated the items of the tasks as either 4 or 5 and the total number of experts involved in validation ( Polit & Beck, 2006). Scale Content Validity Index (S-CVI/Ave) was also calculated by taking the average of I-CVI across each task. The test-retest reliability was established using the Intra class correlation coefficient (ICC) analysis at 95% confidence interval with absolute agreement using two-way mixed model.

The responses obtained from the assessment of reasoning skills using the three tasks were analyzed quantitatively and qualitatively. For the quantitative analysis, the responses across the explanation, prediction, and justification components of the inference tasks were scored based on their appropriateness. The appropriateness of the answers was determined based on the answer key prepared by the researchers, considering logic and rationality. Children received a score of ‘one’ for the appropriate reasons and ‘zero’ for the inappropriate or no reasons for all the tasks. For example, for an explanation based question, ‘Why did Virat get scared?’, if the child’s response was ‘because he saw the dog’ or ‘because the dog would bite,’ it was interpreted as appropriate and received a score of ‘one.’ Whereas if the child’s response to the same question was unrelated (such as ‘because he saw a ghost’ or ‘don’t’ know’), then such answers were considered inappropriate and scored ‘zero’ accordingly. The closed-end polar questions (Yes/No type) of the inference based reasoning task were scored as ‘one’ for the correct answers and ‘zero’ for the incorrect answers. A child could thus obtain a maximum score of ‘six’ for each reasoning task. To prevent bias in the scoring, two independent researchers who were blind to the participants’ demographic details scored the responses, and any disagreements were resolved by reaching a consensus through discussion with a third independent researcher. The scored data were subjected to one-way ANOVA with post hoc Tukey test using SPSS V.25 software to investigate the quantitative changes in reasoning skills with age during the preschool period. Receiver Operating Characteristic (ROC) analysis was also carried out to determine the cut-off values distinguishing the significant quantitative changes in reasoning skills revealed during ANOVA analysis. Gender wise analysis of the task scores was carried out using Independent t test.

The data was further analyzed qualitatively, exploring the manner in which children reason with increase in age to get an insight into the typical pattern of reasoning during the preschool years. Such an analysis was expected to serve as a guide for stimulating typical reasoning development in clinical and educational settings. A general inductive approach ( Thomas, 2006), a systematic set of procedures for analyzing qualitative data that produce reliable and valid findings, was followed for qualitative analysis of the reasoning responses. Two independent researchers coded and categorized the responses for explanation, prediction, and justification of the inference tasks. The codes and categories obtained from the two researchers were compared and merged into a combined set to establish the extent of overlap. The agreement between the two coders was set at 95%, and disagreements were resolved through discussion with a third independent researcher. Based on this process and the consensus among the researchers, the responses of the participants were qualitatively categorized into ‘Appropriate reasoning with story content,’ ‘Appropriate reasoning integrating story content with prior knowledge,’ ‘Inappropriate reasoning with improper story content usage,’ and ‘Inappropriate reasoning with context associated with prior knowledge but improper to the story content’ and ‘No reason.’ An example of the qualitative analysis of one such response from the first section of the story is provided in Table 1. For more details of the associated data see underlying data ( Prasanna *et al.*, 2023a).

**Table 1.** Examples of the data analysis based on story section 1.

Story	Reasoning tasks		Reasoning response analysis				
Theme	Tasks	Question	Appropriate reasons Score: 1		Inappropriate reasons Score: 0		No reasons Score: 0
			Appropriate reasoning with story content	Appropriate reasoning integrating story content with prior knowledge	Inappropriate reasoning with improper story content usage	Inappropriate reasoning with context associated with prior knowledge but improper to the story content	No reasons
A boy visiting his grandparent's house gets scared of seeing a dog, and the dog is locked in the cage later.	Explanation	**For example,** Why did Virat get scared?	**For example,** Because he saw the dog	**For example,** Because Dog will bite	**For example,** Scooter	**For example,** He saw ghost	**For example,** Don’t know
	Prediction	**For example,** What would have happened if grandpa hadn't locked the dog in the cage?	**For example,** The boy will keep crying	**For example,** Dog will come and bite	**For example,** Locked the dog	**For example,** Dog will fall down	**For example,** Don’t know
	Inference	Did Virat like his grandparents? (Inference)	**For example:** Y **es**		**For example:** No		**For example,** Don’t know
		What made you feel so? (Justification of inference)	**For example,** Because he hugged grandparents	**For example,** Because I love my grandparents	**For example,** Dog barked	**For example,** He doesn’t like the car	**For example,** Don’t know

## Results

The story-based tasks for assessing reasoning skills in preschoolers were developed. The details regarding of finalized story and the tasks are shown in Table 2. The psychometric properties of the tasks were determined using content validity and test-retest reliability measures. The content validity measures indicated appropriate validity as all the items of the reasoning tasks obtained an I-CVI of 0.83 or higher and S-CVI of 0.98 ( Almanasreh *et al.*, 2019; Polit & Beck, 2006). The test-retest reliability measures computed using ICC at 95% confidence interval with absolute agreement using two-way mixed model revealed an ICC of 0.76, indicating moderate test-retest reliability.

**Table 2.** Details of story and reasoning tasks with examples.

Section	Story	Number of pictures	Number of story elements	Reasoning Tasks		
				Explanation (Open-ended questions)	Prediction (Open-ended questions)	Inference (Closed-ended yes/no question and open-ended question)
**S1**	Virat was a 6-year-old boy. On a week day, Virat’s parents dropped him at his grandparents’ house in a scooter. Virat’s grandparents were waiting for him at the gate. Virat ran towards his grandparents and hugged them. Suddenly, Virat saw a dog coming towards him barking. Virat got scared and started crying. His grandpa pulled and locked the dog in the metal cage. After this, Virat entered the house happily with grandparents.	2	14 **For example,** Grandparents were waiting at the gate	2 **For example,** Why did Virat get scared?	2 **For example,** What would have happened if grandma hadn't locked the dog in the cage?	2 **For example,** Did Virat like his grandparents? (Inference) What made you feel that way? (Justification of inference)
**S2**	It was raining. Hence, grandpa told Virat not to play outside. Virat looked through the window. He saw the dog in the cage getting wet. Water was leaking through a hole on its roof. Dog was sleeping at the corner. Seeing this, Virat took umbrella from cupboard and ran to the cage. He kept umbrella above the cage. Then water stopped leaking. Dog started wagging tail. Seeing this, Virat started running to the house. Suddenly Virat heard a strange sound from the side of the house.	2	14 **For example,** He kept umbrella above the cage	2 **For example,** Why did Virat keep an umbrella on the cage?	2 **For example,** What would have happened if Virat had played in the rain?	2 **For example,** Did Virat help the dog? (Inference) What made you feel that way? (Justification of inference)
**S3**	Virat ran towards the side where the sound came. A robber wearing a black mask caught Virat and closed his mouth. Seeing this, the dog started barking loudly. Everybody came outside the house and the robber ran away. Virat slowly went to the cage and tried to touch the dog. The dog started licking his hand. Virat asked grandpa to open the cage and the dog came out happily. Virat gave biscuits to the dog. They became friends.	2	14 **For example,** Dog started barking loudly	2 **For example,** Why did the dog bark loudly?	2 **For example,** What would have happened if Grandparents were not at home when the robber came?	2 **For example,** Did the Grandparents catch the robber? (Inference) What made you feel that way? (Justification of inference)

**Figure 1.**
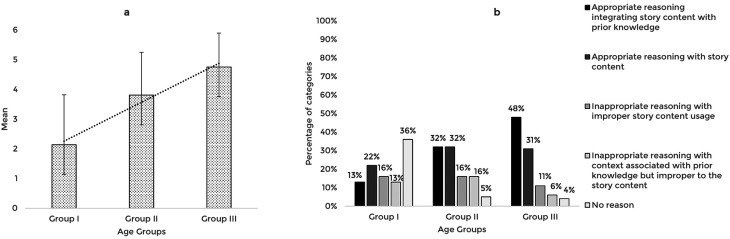
(a) Mean and standard deviation of the reasoning scores and (b) the percentage of categories of reasoning responses for the explanation task across the age groups.

**Figure 2.**
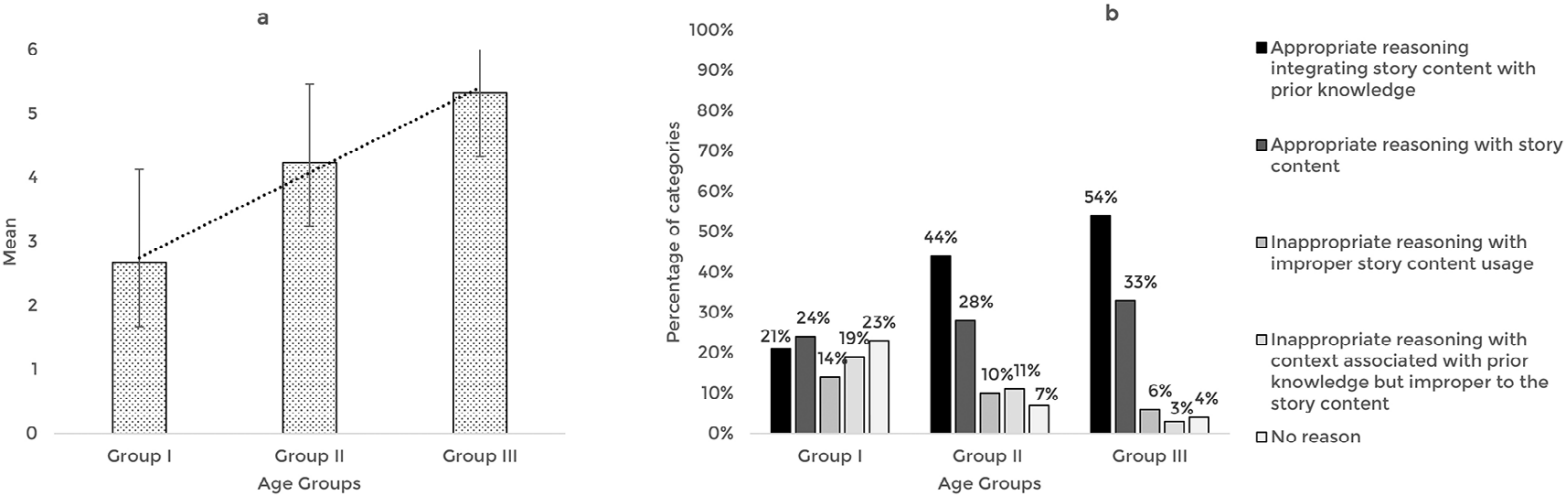
(a) Mean and standard deviation of the reasoning scores and (b) the percentage of categories of reasoning responses for the prediction task across the age groups.

**Figure 3.**
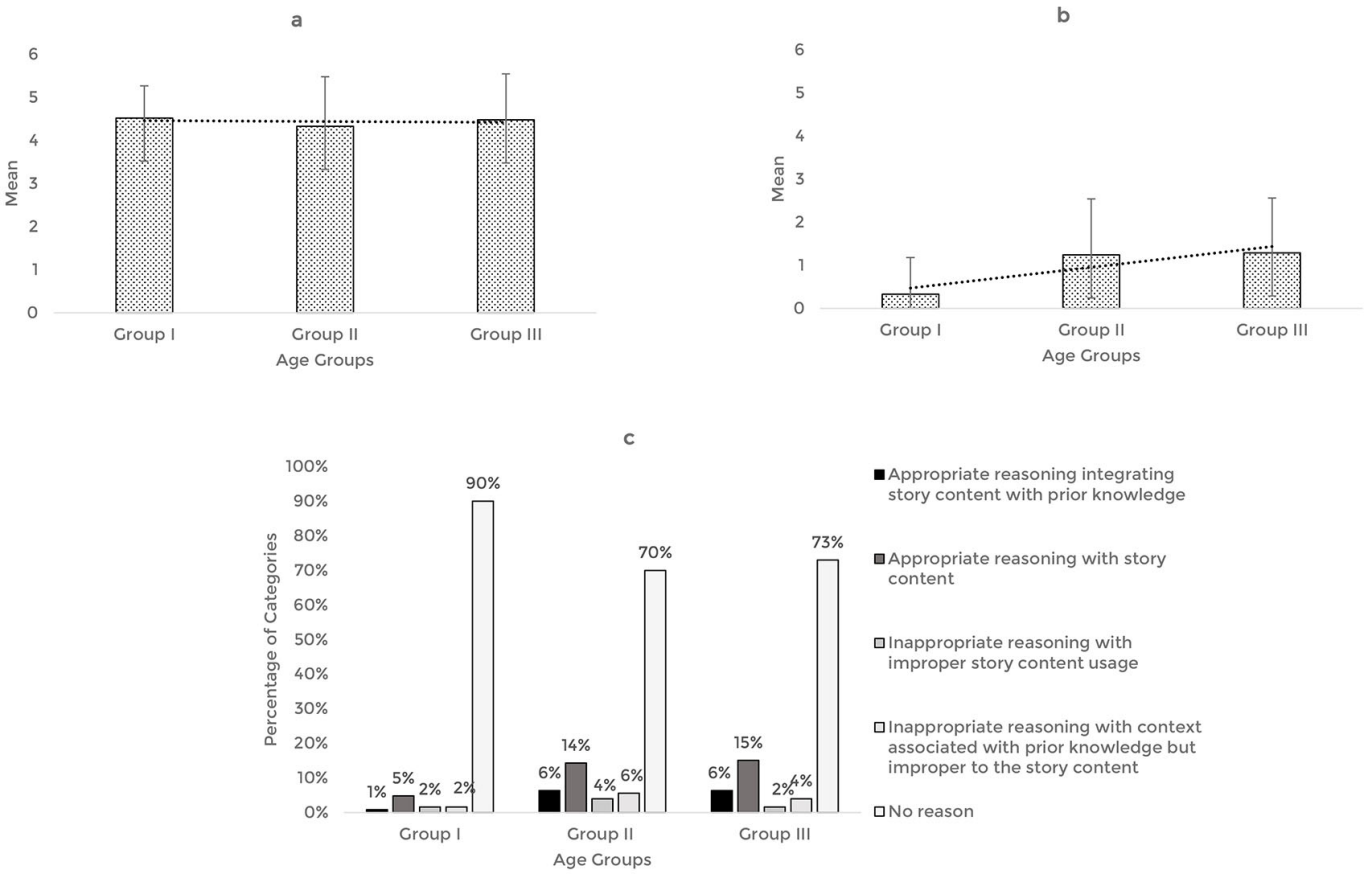
(a) Mean and standard deviation of the reasoning scores under inference, (b) justification of inference, and (c) the percentage of categories of reasoning responses for the justification of inference task across the age groups.

The validated story-based tasks were then used to assess the reasoning abilities of 63 typically developing Indian preschool children. The responses obtained from the participants across the explanation, prediction, and inference based reasoning tasks were quantitatively and qualitatively analyzed. The mean and standard deviation of the total reasoning scores and the percentage of categories of responses for the explanation, prediction, and inference based reasoning tasks across the age groups are illustrated in Figure 1(a,b), Figure 2(a,b), and Figure 3(a-c) respectively.

A comparison of the mean scores of the explanation based reasoning tasks revealed that the explanation based reasoning scores increased with age, F (2,60)=17.91, p<0.001, irrespective of the gender (t[61]=0.401,p=0.690). The post hoc pairwise comparison showed that the older groups (Groups II and III) performed significantly better (p<0.01) than the younger group (Group I); however, there was no statistically significant difference between the older groups (p=0.08). Receiver Operating Characteristic (ROC) analysis revealed a cut-off score of 2.5 discriminating between the younger (Group I) and older groups (Groups II and III) for explanation-based reasoning performance with 81% sensitivity, 62% specificity, and 0.8 Area under the curve (AUC) that is statistically significant (p<0.05).

The qualitative analysis using the percentage of categories of reasoning responses showed that with the increase in age, most children reasoned appropriately, integrating story content with prior knowledge. The codes identified under appropriate reasoning categories and their frequency across the age groups under the explanation task are presented in Table 3.

**Table 3.** Codes with its frequencies under appropriate reasoning categories for explanation task.

Question	Group I Codes (Frequency)	Group II Codes (Frequency)	Group III Codes (Frequency)
Explanation Q1	Dog (10) Dogs action (1)	Dog (9) Dogs action (6)	Dog (16) Dogs action (2)
	-	-	Bite (2) Opened and kept dog (1)
Explanation Q2	To see dog (2) To see grandparents (1)	To see dog (2)	To see dog (2)
	To study/Play (2) Parents have to go out (3) Virat doesn't know the way (1)	To study/Play (1) Parents have to go out (4) Virat doesn't know the way (1) Grandparents missed him (1) Wanted to stay (1) Naughty (2) Safety (2)	To study/play (3) Parents have to go out (6) Virat doesn't know the way (1) Grandparents missed him (1) Likes grandparents (1) Wanted to stay (1) Naughty (1)
Explanation Q3	Rain (5)	Rain (7)	Rain (4) Dog wet (2)
	Good boy (2) Prevent wetting (3) Dog wanted (1)	Prevent wetting (7) Rainfall on dog (1)	Prevent wetting (9) Prevent being ill (2)
Explanation Q4	Kept Umbrella (1)	Kept Umbrella (3)	Kept Umbrella (2)
	-	Wanted (1) Like (2) Wind (1)	Like (6) Happy (1) Thanks for help (1)
Explanation Q5	Robber (3) Robber caught and closed mouth (4)	Robber (4) Robber caught and closed mouth (7)	Robber (4) Robber caught and closed mouth (7)
	Robber takes away the boy (1) To come out of cage (1)	Robber takes away the boy (1) To come out of cage (1) Dog loved the boy (1) Save boy (1)	Robber takes away the boy (2) To come out of cage (1) Save boy (6)
Explanation Q6	Since boy was crying (1)	Boy went there (1) Since boy went outside (1)	
	To take boy and go (1) For not to scream (1) Not to call grandparents (1)	Stop breathing (1) All not to listen (2) For not to scream (6) Since boy went there alone (1) Police not to come (1) To die (1)	For not to scream (9) Stop breathing and lose consciousness (1) All not to listen (3) Not to call grandparents (1) To take boy and go (2)

Note: The writing on white background represents codes identified under the category of ‘appropriate reasoning with story content.’ The writings under grey background represent the codes identified under ‘appropriate reasoning integrating story content with prior knowledge’.

The comparison of the mean scores for the prediction based reasoning task also showed a significant increase with age F (2,60)=26.58, p<0.001, irrespective of the gender t (61)=0.564, p=0.575. Post hoc pairwise comparison revealed a significant difference in prediction scores between all three age groups (p<0.01). The ROC analysis revealed a cut-off prediction score of 4.5 discriminating between Group I and Group II with 52% sensitivity, 90% specificity, and 0.8 statistically significant AUC (p<0.001), and a cut-off prediction score of 5.5 discriminating Group II from Group III with 52% sensitivity, 86% specificity, and 0.7 AUC, that is statistically significant (p<0.05).

The qualitative analysis showed that similar to the explanation task, most children reasoned integrating story content with prior knowledge as their age increased. The codes identified under appropriate reasoning categories and their frequency across the age groups under the prediction task are presented in Table 4.

**Table 4.** Codes with its frequencies under appropriate reasoning categories for prediction task.

Question	Group I Codes (Frequency)	Group II Codes (Frequency)	Group III Codes (Frequency)
Prediction Q1	Enter home (1) Walk and go (1)	Will not run (2) No cry (1)	Walk and go (2) Will not run (1) Enter home (2) No cry (1) Stand there itself (1)
	Will bite (3) Touch the dog (2) Make noise (1)	Happy (3) Touch the dog (2) Dog will catch the boy (1) Will play (1) Loved the dog (1) Will not bite (1) Will bite (1)	Walk and go nicely (1) Brave (4) Will play (2) Will bite (1) Touch the dog (1) Loved the dog (1) Food to dog (1)
Prediction Q2	Run and go (3) Scared (1) Run and came (1)	Scared (4) Run and come (1) Bark (4)	Scared (1) Stand there itself (1) Run and go (1) Boy cry (2)
	Dog cry (1) Bite (5) Go out (2)	Bite (5) Jump on him (1) Will not do anything (1) Go out (2)	Go out (4) Bite (10)
Prediction Q3	Wet (5) Rainfall (3)	Wet (6) Rainfall (1)	Wet (6)
	Ill with fever or cold (6) Thunder and lightning will kill (1)	Ill with fever or cold (9) Wet and mother shout (1) Fall (1) Ask grandpa umbrella (1)	Dog will make sound (1) Fall (1) Ill with fever or cold (13)
Prediction Q4	Rainfall (6) Wet (7)	Run inside (1) Wet (8) Rainfall (2) Call grandpa (1)	Wet (9) Not able to keep umbrella (1) Rainfall (2) Cry (1)
	Find out (1)	Raincoat (5) Cold (1) Cage inside (1)	Wouldn't go (2) Fever (1) Raincoat (4) Sad (1)
Prediction Q5			Bark (2)
	Robber will throw (1) Take and go (2) Die (1)	Take and go (11) Robber will take items from home (1) Danger (1) Robber put boy in jail (2)	Take and go (15) Dog will inform barking, and they will come from back (1)
Prediction Q6	Will not catch (1) Robber wouldn't have come (1)	Will not catch (3) Will not bark (1)	Will not catch (9)
		Danger (1)	Play (2) Will catch when went to Play with dog (1) Escape (1) Dog will bark and thus will get to know (1) Ran away (1)

Note: The writing on white background represents codes identified under the category of ‘appropriate reasoning with story content.’ The writings under grey background represent the codes identified under ‘appropriate reasoning integrating story content with prior knowledge’.

For the inference-based reasoning tasks, comparison of the mean score revealed that the inference scores remained almost similar across the age groups, F (2,60)=0.206, p=0.82, irrespective of the gender (t [61]=0.167, p=0.868). However, the scores pertaining to the justification for inference were found to increase with age, F (2,60)=6.04, p<0.05, without gender as a factor (t [61]=1.767, p=0.082). Post hoc pairwise comparison revealed significant differences in the inference justification performance between older groups (Groups II and III) and younger group (Group I) (p<0.05). There was no statistically significant difference between the older groups (Group II and III) (p=0.99). ROC analysis determined the cut-off score as 0.5, discriminating between the younger (Group I) and older groups (Groups II and III) for inference justification scores with 62% sensitivity, 86% specificity, and 0.7 statistically significant AUC (p<0.05).

Qualitative analysis showed that most of the responses belong to the ‘No reason’ category across the age groups and a smaller percentage of responses under ‘appropriate reasoning’ categories. Among the ‘appropriate categories,’ majority of the preschoolers reasoned under the category of ‘appropriate reasoning with story content.’ The codes identified under the smaller percentage of appropriate reasoning categories and their frequency across the age groups under the justification of inference task are presented in Table 5.

**Table 5.** Codes with its frequencies under appropriate reasoning categories for justification of inference task.

Question	Group I Codes (Frequency)	Group II Codes (Frequency)	Group III Codes (Frequency)
Justification for Inference Q1	-	-	-
Justification for Inference Q2	-	Hugged (1) When dog came to bite (1) Granny locked the dog (1)	-
	I love (1)	I love (1)	Saw love (1)
Justification for Inference Q3	Kept umbrella (2) Umbrella (1)	Kept umbrella (9)	Kept umbrella (7)
	-	Helped by keeping umbrella (1)	Boy couldn't afford dog getting wet so kept umbrella (1)
Justification for Inference Q4	Went outside while raining (1)	-	Told not to go outside (1) Told to take care when going outside (1)
	-	Alone (2) Mother takes care of me (1)	Grandpa tried not to bite (1)
Justification for Inference Q5	Licking hands (1)	Opened dog (1) Licking hands (1) Robber caught and Dog barked (2) Went to the cage (1)	Licked hands (2) Dog made robber ran (2) When barked (2) Touched (1)
	-	Dog helped (1) Loved dog (1)	Dog helped (2) Saved him (1)
Justification for Inference Q6	Robber ran (1)	Ran away (1)	Robber ran (3)
	-	Robber ran fast (1)	Grandparents at house (1) Robber ran and should have called police (1)

Note: The writing on white background represents codes identified under the category of ‘appropriate reasoning with story content.’ The writings under grey background represent the codes identified under ‘appropriate reasoning integrating story content with prior knowledge’.

## Discussion

The evaluation of psychometric properties of the story-based reasoning tasks revealed adequate content validity and test-retest reliability. The developed stimuli consisting of a story and reasoning tasks in a question format align with the theoretical framework proposed for exploring reasoning skills among preschoolers ( Kendeou *et al.*, 2019). The use of story as the assessment material is also consistent with the other research that supports the efficacy of story-based approaches among preschoolers ( Dawes *et al.*, 2019; O’Reilly *et al.*, 2022). Besides adding culturally and linguistically relevant data on reasoning skills to the existing literature in this domain among preschoolers ( Muller *et al.*, 2007; Shtulman & Carey, 2007), the current study has offered additional depth by exploring the development of reasoning skills across explanation, prediction and inference domains using a single story. Moreover, the present format of these tasks, wherein domains of the reasoning abilities could be assessed on spontaneously emerged output of typically developing preschoolers, is aligned with the earlier perspectives in this regard ( Reed *et al.*, 2015). Further, the components of the story, such as the characters, sequences, objects, dress, pictures, vocabulary, and task formats, were designed to reflect the real-life Indian context, which could offer appropriate familiarity and suitability to Indian preschoolers and aid in accurate representation of their abilities ( Rao *et al.*, 2021; Sternberg, 2004).

The developed story-based tasks assessed the reasoning abilities of typically developing Indian preschool children. The comparison of mean reasoning scores across the age groups in the explanation, prediction, and justification of inference task revealed that the preschooler’s reasoning skills improved significantly with age. In most tasks (explanation and justification of inference), a significant change in performance was observed from Group I (36–48 months) to Group II (49–60 months) and III (61–72 months) than between Group II (49–60 months) and III (61–72 months), indicating the rapid development of reasoning skills during the initial phase of the preschool period. The results agree with the previous literature that younger preschoolers show a significant improvement in reasoning skills with age than older preschoolers ( Hong *et al.*, 2005). The results also imitate the age bands of thinking differences (Symbolic function substage: 2–4 years, Intuitive thought substage: 4 to 7 years) in Piaget’s preoperational stage of cognitive development ( Piaget, 1952). However, the results obtained in the prediction task showed evidence for age-wise developmental changes than in age bands, with significant improvement in performance with age in years. The improved performance in reasoning skills with age across the tasks was not surprising as the preschool period is known to be critical for most of the developmental domains, including executive function skills ( Garon *et al.*, 2008).

Contrary to the expectation, the age-related difference in reasoning performance was not found in the inference task. More specifically, the findings from the inference task suggested that Group I (36–48 months) children performed like the Group II (49–60 months) and III (61–72 months) children. These results indicate that children as young as 36–48 months of age (Group I) can make inferences. Previous literature supports the findings attesting that preschoolers make logical inferences easily and earlier in development ( Moshman, 2004). However, we could not exclude the fact that the inference task’s yes/no question nature might also have influenced the extent of this lack of age-related differences. In yes/no questions, researchers opine that younger preschoolers might show a yes/no bias (all yes responses, i.e., yes bias and vice versa) due to social pressure or difficulty inhibiting yes responses ( Okanda & Itakura, 2010). Therefore, there is a chance that the younger participants in this study might have also undergone such yes/no bias in the inference task influencing the lack of age-related differences. Hence, it indicates that, though yes/no questions have been recommended for reasoning skills assessment ( Säre *et al.*, 2017), we need to be cautious regarding the chance of yes/no bias in younger preschoolers. However, despite the probability of yes/no bias in this study, the preschooler’s early inferential ability observed in the previous literature adds substantial weight to the conclusion of inferential ability in younger preschoolers.

Another interesting finding comparing mean reasoning scores was that the preschoolers performed poorly on the justification of inference task than on the other tasks. Though preschoolers could make inferences, the findings contend that they find it difficult to justify their inferences. This difficulty could be because the ability to justify the inferences is a higher quality of thinking requiring metacognition ( Moshman, 2004). Though metacognition was not explicitly studied in this research, previous literature findings indicate that children require metacognitive awareness to justify their inferences ( Whittaker & McMullen, 2014), which is beyond the preschool age in development ( Veenman *et al.,* 2006), lend support for the poorer performance in the current study. However, it is also noteworthy that a smaller percentage of older preschoolers (Group II, 49–60 months and Group III, 61–72 months) could provide appropriate responses (20%) in the justification of inference tasks using story content. Though it could be due to individual differences, it might also indicate the traces of metalogical development for reasoning from 4 years of age using the linguistic content, suggesting the scope of improvement in justification if provided with support.

On qualitative analysis, results from the explanation and prediction task revealed that among the appropriate reasons made by the preschoolers, younger preschoolers (Group I, 36–48 months) predominantly reason under the category of ‘reasoning with story content,’ and the older preschoolers (Group II, 49–60 months and Group III, 61–72 months) reason in the category of ‘reasoning integrating story content with prior knowledge.’ The results indicate that while younger preschoolers reason with reference to the inference based on the story content premise, older preschoolers undergo a higher thinking process of coordinating the logical inferences from the story and prior knowledge to conclude and reason. The results support the previous literature findings that preschoolers integrate inferences from linguistic context ( Florit *et al.*, 2011) and access prior knowledge while reasoning ( Gopnik *et al.*, 2004). The findings also align with the comprehension development process, which activates and integrates linguistic knowledge with background knowledge to connect the story contents and form a mental representation to comprehend ( Kendeou *et al.*, 2019).

Another interesting finding in qualitative analysis was with respect to the justification of the inference task. The results from the justification of inference task revealed that preschoolers depend on the source of knowledge from the linguistic contents to justify their inference. We have observed that older preschoolers tried to justify inference with respect to the source of knowledge from the story contents rather than the prior knowledge premise. It was also noted that most preschoolers provided empty reasons such as ‘I know’ for justification questions, claiming they had always known the inference. The findings are supported by the previous literature ( Kuhn *et al.*, 2004; Taylor *et al.*, 1994) that children tend to give such responses when unsure or unaware of the answer. This could be because the metacognitive awareness of reasoning is in the emerging stage during the preschool period ( Veenman *et al.*, 2006). Based on the pattern of development observed in other tasks of reasoning, it could be expected that with increase in age over the preschool period, with the development of metacognitive awareness of reasoning, children might justify their inference majorly by integrating the story content with their prior knowledge and own interpretation, thereby following the similar pattern of reasoning development.

All the developmental changes in reasoning obtained in this study could be attributed to the biological maturational change of the brain and reconfiguration of frontoparietal networks ( Andrews, 2020) and facilitated by the everyday interactional context ( Niklas *et al.*, 2018; Säre, Luik, & Tulviste, 2016b), working memory ( Andrews, 2020), language ( Richland & Burchinal, 2013) and theory of mind development ( Taggart *et al.*, 2005; Whittaker & McMullen, 2014). Consistent with existing literature gender wise differences were not found in all the reasoning tasks ( Ardila *et al.*, 2011). The study’s findings need to be cautiously generalized, as reasoning responses are dynamic.

In conclusion, the study describes the general reasoning skill development in typically developing Indian preschool children between 36 to 72 months of age using story-based tasks. The study put forth a valid and reliable story-based task for the purpose, and the assessment concluded that reasoning skills increase with age during preschool in a pattern of moving from reasoning based on story content to reasoning integrating the story content with prior knowledge. The study findings provide clinical and educational implications with insights on general reasoning development in preschoolers and the utility of age-appropriate story-based explanation, prediction, and inference question tasks to assess and foster reasoning responses in preschoolers. The findings also guide educators to scaffold reasoning in preschoolers, and the developed stimuli aid in early identification of reasoning deficits and guide planning intervention for Indian preschoolers with reasoning deficits. Though the study findings provide insight into general reasoning development in preschoolers, the role of contributing factors such as working memory, theory of mind, and social interaction was not explored, and it remains a limitation of the present study. Therefore, future studies considering such factors could provide an in-depth understanding of reasoning development to assess and foster reasoning during preschool years.

## Data Availability

Figshare: ‘An Insight into developmental changes in reasoning skills among Indian Preschoolers - A Cross-Sectional Study using a story-based approach’, https://doi.org/10.6084/m9.figshare.22140617.v1 ( Prasanna *et al.*, 2023a). This project contains the following underlying data:
• Data file 1. (Quantitative and Qualitative data of Reasoning responses) Figshare: STROBE checklist for ‘An Insight into developmental changes in reasoning skills among Indian Preschoolers - A Cross-Sectional Study using a story-based approach’, https://doi.org/10.6084/m9.figshare.22140689.v2 ( Prasanna *et al.*, 2023b). Data are available under the terms of the Creative Commons Attribution 4.0 International license (CC-BY 4.0).
