# Supplementary material for: An Insight into developmental changes in reasoning skills among Indian Preschoolers: A cross-sectional study using a story-based approach
Source: F1000Res. 2024 Oct 11;12:446. Originally published 2023 Apr 26. [Version 3] doi: 10.12688/f1000research.131906.3 (PMC11582391; doi:10.12688/f1000research.131906.3)
Supplement: Peer review history

# Reviewer report: Reviewer response for version 3

**DOI:** 10.5256/f1000research.172849.r357827

**Published:** 2025-02-04

**Authors:** Tulika Borah

**Affiliations:** 1 Assam Agricultural University, Jorhat, Assam, India

This study offers an insightful exploration of developmental changes in reasoning skills among Indian preschoolers, utilizing a story-based approach. The cross-sectional design is appropriate for capturing key age-related differences in reasoning abilities at different stages of early childhood. The use of stories as a tool to assess cognitive development is particularly engaging, as it reflects how young children interact with and process information in a naturalistic setting.

 The findings contribute valuable knowledge to the growing body of literature on early cognitive development, specifically in the Indian context. However, there is room for further discussion on the cultural relevance of the stories used and how they may influence reasoning patterns across different regions or socioeconomic backgrounds in India. A deeper consideration of these cultural nuances could strengthen the generalizability of the results.

 The methodology is robust, but a longitudinal design could provide a more comprehensive understanding of how reasoning skills evolve over time. Additionally, a more detailed exploration of individual differences, such as socio-economic status, family environment, or educational exposure, level and type of intelligence could offer a fuller picture of the factors that shape cognitive development in Indian preschoolers.

 Overall, the article is well-written and offers important insights into early childhood reasoning skills. It would benefit from further contextual analysis and exploration of broader factors influencing cognitive development.

 Suggested editing

 Section- Discussion:

 Para 2, 1 ^st^ line- ‘The tool utilized that consisted of a story and reasoning tasks in a question format aligned with the theoretical framework proposed for exploring reasoning skills among preschoolers’

 Para 9, 9 ^th^ line- ‘Though the study findings provide insight into general reasoning development in preschoolers, the sampler size was smaller and the role of contributing factors such as working memory, theory of mind, language, bilingualism/multilingualism, and social interaction on reasoning was not explored, and it remains a limitation of the present study’.

Is the work clearly and accurately presented and does it cite the current literature?

Yes

If applicable, is the statistical analysis and its interpretation appropriate?

Yes

Are all the source data underlying the results available to ensure full reproducibility?

Yes

Is the study design appropriate and is the work technically sound?

Yes

Are the conclusions drawn adequately supported by the results?

Yes

Are sufficient details of methods and analysis provided to allow replication by others?

Yes

Reviewer Expertise:

Early childhood Care and Education, Spatial intelligence

I confirm that I have read this submission and believe that I have an appropriate level of expertise to confirm that it is of an acceptable scientific standard.

# Reviewer report: Reviewer response for version 3

**DOI:** 10.5256/f1000research.172849.r330866

**Published:** 2024-11-21

**Authors:** Ramesh Kaipa

**Affiliations:** 1 Department of Communication Sciences and Disorders, Oklahoma State University, Stillwater, Oklahoma, USA

I enjoyed reading the resubmission. I recommend this article for approval.

Is the work clearly and accurately presented and does it cite the current literature?

Partly

If applicable, is the statistical analysis and its interpretation appropriate?

Yes

Are all the source data underlying the results available to ensure full reproducibility?

Yes

Is the study design appropriate and is the work technically sound?

Yes

Are the conclusions drawn adequately supported by the results?

Partly

Are sufficient details of methods and analysis provided to allow replication by others?

Yes

Reviewer Expertise:

Speech and Language Therapy

I confirm that I have read this submission and believe that I have an appropriate level of expertise to confirm that it is of an acceptable scientific standard.

# Reviewer report: Reviewer response for version 2

**DOI:** 10.5256/f1000research.168597.r300709

**Published:** 2024-08-27

**Authors:** Ramesh Kaipa

**Affiliations:** 1 Department of Communication Sciences and Disorders, Oklahoma State University, Stillwater, Oklahoma, USA

I thank the authors for addressing my comments. My only comment is that considering a moderate test-retest reliability (0.66), how can authors explain the reliability of the findings? Otherwise, this paper is a good addition to the existing literature.

Is the work clearly and accurately presented and does it cite the current literature?

Partly

If applicable, is the statistical analysis and its interpretation appropriate?

Yes

Are all the source data underlying the results available to ensure full reproducibility?

Yes

Is the study design appropriate and is the work technically sound?

Yes

Are the conclusions drawn adequately supported by the results?

Partly

Are sufficient details of methods and analysis provided to allow replication by others?

Yes

Reviewer Expertise:

Speech and Language Therapy

I confirm that I have read this submission and believe that I have an appropriate level of expertise to confirm that it is of an acceptable scientific standard, however I have significant reservations, as outlined above.

# Response

**Published:** 2024-10-01

**Authors:** Gagan Bajaj

**Affiliations:** Kasturba Medical College, Mangalore, India

1. **I thank the authors for addressing my comments. My only comment is that considering a moderate test-retest reliability (0.66), how can authors explain the reliability of the findings? Otherwise, this paper is a good addition to the existing literature.**

**Reply:** We thank the reviewer for highlighting this aspect and giving us the opportunity to clarify. We have discussed the moderate test-retest reliability of our task in relation to other similar cognitive tasks. Furthermore, we have provided a potential explanation for these findings and outlined future directions for further optimization in the discussion and future directions sections of the manuscript, respectively. We hope the reviewer finds our efforts satisfactory.

# Reviewer report: Reviewer response for version 2

**DOI:** 10.5256/f1000research.168597.r300710

**Published:** 2024-08-23

**Authors:** Andreas Demetriou

**Affiliations:** 1 University of Nicosia, Nicosia, Nicosia, Cyprus

The paper is sufficiently improved so that it can be accepted for indexing.

Is the work clearly and accurately presented and does it cite the current literature?

Yes

If applicable, is the statistical analysis and its interpretation appropriate?

Yes

Are all the source data underlying the results available to ensure full reproducibility?

Yes

Is the study design appropriate and is the work technically sound?

Partly

Are the conclusions drawn adequately supported by the results?

No

Are sufficient details of methods and analysis provided to allow replication by others?

Yes

Reviewer Expertise:

Cognitive development and individual differences in intelligence. Integration of cognitive developmental and psychometric theory.

I confirm that I have read this submission and believe that I have an appropriate level of expertise to confirm that it is of an acceptable scientific standard.

# Response

**Published:** 2024-10-01

**Authors:** Gagan Bajaj

**Affiliations:** Kasturba Medical College, Mangalore, India

**1. The paper is sufficiently improved so that it can be accepted for indexing.**

**Reply:** We are grateful to the reviewer for accepting the paper for indexing.

# Reviewer report: Reviewer response for version 1

**DOI:** 10.5256/f1000research.144794.r206526

**Published:** 2023-11-21

**Authors:** Andreas Demetriou

**Affiliations:** 1 University of Nicosia, Nicosia, Nicosia, Cyprus

The study is interesting and the paper well written. It has several limitations:

1. The number of children in each group is small. Power must be reported.
2. No specification of logical schemes is made.
3. No differentiation between changes in reasoning and language. No association with brain is justified.

Is the work clearly and accurately presented and does it cite the current literature?

Yes

If applicable, is the statistical analysis and its interpretation appropriate?

Yes

Are all the source data underlying the results available to ensure full reproducibility?

Yes

Is the study design appropriate and is the work technically sound?

Partly

Are the conclusions drawn adequately supported by the results?

No

Are sufficient details of methods and analysis provided to allow replication by others?

Yes

Reviewer Expertise:

Cognitive development and individual differences in intelligence. Integration of cognitive developmental and psychometric theory.

I confirm that I have read this submission and believe that I have an appropriate level of expertise to confirm that it is of an acceptable scientific standard, however I have significant reservations, as outlined above.

# Response

**Published:** 2024-06-28

**Authors:** Gagan Bajaj

**Affiliations:** Kasturba Medical College, Mangalore, India

**1. The study is interesting and the paper well written. It has several limitations.**

**Reply: **We are grateful to the reviewer for the constructive feedback and suggestions. The authors have endeavoured to revise the manuscript according to these valuable suggestions and hope that the reviewer will find our efforts satisfactory.

**2. The number of children in each group is small. Power must be reported.**

**Reply: **The sample size of the study was determined using the formula n = Z _1-α/2_^2 ^SD ^2 ^/d ^2^ at 95% confidence level, 20% absolute error, 80% power with an anticipated standard deviation of 0.789 based on pilot testing. The same has been reported in the method section.  The present study was initiated as a preliminary investigation of a larger project aimed at exploring cognitive-communication skills within this population. Undoubtedly, given its preliminary nature, the study was constrained by a smaller sample size, a limitation that has been transparently acknowledged and addressed within the study's limitations section.

**3. No specification of logical schemes is made.**

**Reply:** This research serves as a continuation of our previous work, which developed a tool specifically designed for assessing cognitive-communication skills among preschoolers in the Indian context, based on a story (Prasanna, Bajaj, et al., 2024). The current study represents the evaluation phase of this tool, focusing on the reasoning development among Indian preschoolers. The manuscript has been updated to clearly emphasize this aspect. Additionally, we have provided sub-headings in the method section to enhance the logical flow of the information.

 4. **No differentiation between changes in reasoning and language.**

**Reply:**  Thank you for the kind feedback. We acknowledge that the present study exclusively included children with age-appropriate speech and language abilities, with their responses analysed for cognitive accuracy. While we recognize the potential for analysing responses in terms of various linguistic elements, such as syntactic and semantic depth, this aspect was beyond the scope of the present research. For example, in a task involving a reasoning question like "Why did Virat get scared?" responses such as "Dog," "When saw dog," and "Because dog was there" were all deemed correct reasoning responses, despite their linguistic differences in Mean Length of Utterance (MLU).

 We value the reviewer's suggestion to distinguish between cognitive and linguistic aspects, and we look forward to incorporating this consideration into our future research, particularly in the study of reasoning abilities among children with language disorders. Same has been updated under the future directions of the present study.

 5. **No association with brain is justified.**

**Reply:** Thank you for the kind suggestion. The neurophysiological correlates of reasoning abilities among preschoolers, discussed in the present work, have been updated in the discussion section.

# Reviewer report: Reviewer response for version 1

**DOI:** 10.5256/f1000research.144794.r170971

**Published:** 2023-07-05

**Authors:** Ramesh Kaipa

**Affiliations:** 1 Department of Communication Sciences and Disorders, Oklahoma State University, Stillwater, Oklahoma, USA

Summary: This is a well-developed study. The authors have attempted to develop a stimulus that is culturally relevant to Indian children. The authors need to be appreciated for this line of work. I do have some comments that are mentioned below.

**Introduction:**

1. The rationale for this study is not developed appropriately. The authors need to justify how will the current line of research advance the empirical knowledge.
2. It would help if the authors could review prior literature pertaining to reasoning skills and language development in children.

**Methods:**

1. “What did the authors mean by “Children who qualified across all the ten items of the TQS”. Is there a cut-off score in TQS? The authors expand on this.
2. Can authors present data on the participants’ language usage? Were they bilingual or multilingual? I believe this variable could skew the findings.
3. The authors mentioned that 13 children were excluded. Was it from the cohort of 63 children?
4. Can I please have the authors present data on the reliability and validity of the assessment tool “A Day at the Grandparents House”?
5. If the story was designed in English, how did the authors make sure that the translated version was faithful to the original version? It would be helpful to compare the psychometric properties of the original version to the translated version.
6. Can the authors present the findings of test-retest reliability for the pilot test on 15 children?

**Results**

1. The results are well presented, along with visual illustrations.

**Discussion**

1. The authors make a case that the findings can be attributed to biological maturation. The authors need to discuss the specific neurophysiological substrates that are correlated with higher-order tasks such as the one used in the current study. 

Is the work clearly and accurately presented and does it cite the current literature?

Partly

If applicable, is the statistical analysis and its interpretation appropriate?

Yes

Are all the source data underlying the results available to ensure full reproducibility?

Yes

Is the study design appropriate and is the work technically sound?

Yes

Are the conclusions drawn adequately supported by the results?

Partly

Are sufficient details of methods and analysis provided to allow replication by others?

Yes

Reviewer Expertise:

Speech and Language Therapy

I confirm that I have read this submission and believe that I have an appropriate level of expertise to confirm that it is of an acceptable scientific standard, however I have significant reservations, as outlined above.

# Response

**Published:** 2024-06-28

**Authors:** Gagan Bajaj

**Affiliations:** Kasturba Medical College, Mangalore, India

**1. Summary: This is a well-developed study. The authors have attempted to develop a stimulus that is culturally relevant to Indian children. The authors need to be appreciated for this line of work. I do have some comments that are mentioned below.**

**Reply: **We are grateful to the reviewer for the constructive feedback and suggestions. The authors have endeavoured to revise the manuscript according to these valuable suggestions and hope that the reviewer will find our efforts satisfactory.

**2. Introduction**

**2a. The rationale for this study is not developed appropriately. The authors need to justify how will the current line of research advance the empirical knowledge.**

**Reply: **The rationale of the study has been revisited to ensure that it aligns with the research gaps illustrated in the introduction and justifies the potential of the present work in advancing empirical knowledge.

**2b. It would help if the authors could review prior literature pertaining to reasoning skills and language development in children.**

**Reply:** The literature on reasoning skills and their significance in children's language development has been reviewed and updated within the manuscript. Consequently, the importance of the association between the two has been underscored in the rationale and discussion sections of the manuscript, respectively.

**3. Methods**

**3a. What did the authors mean by “Children who qualified across all the ten items of the TQS.” Is there a cut-off score in TQS? The authors expand on this.**

**Reply**: Thank you for providing the opportunity to clarify. The TQS does not have any cut-off scoring; instead, it functions as a screening tool for the presence or absence of childhood disabilities. In the present study, the TQS was utilized to ensure that only participants who were found to have no disability across all ten items of the questionnaire were included. This statement has been rephrased in the manuscript to enhance clarity.

**3b. Can authors present data on the participants’ language usage? Were they bilingual or multilingual? I believe this variable could skew the findings.**

**Reply:** The information regarding the number of languages known by the participants in the study has been updated in the manuscript. Although 89% of the participants were bilingual and the remaining 11% multilingual, all of them had English as their L2, with a linguistic proficiency score in English greater than 7 on LEAP-Q (child version). However, since the present study focused solely on the English language, we did not perform an analysis exploring performance differences in the children based on the nature of their L1. This has been noted as a limitation and a future direction in the manuscript.

**3c. The authors mentioned that 13 children were excluded. Was it from the cohort of 63 children?**

**Reply:**  Thank you for providing the opportunity to clarify. A total of 76 children were considered for the study, out of which 13 children were excluded based on the study selection criteria. This left us with 63 children, who were further subdivided into three age groups, each comprising 21 children.

**3d. Can I please have the authors present data on the reliability and validity of the assessment tool “A Day at the Grandparents House”?**

**Reply:**  Thank you for the opportunity to clarify. The tool used in the present research was developed in our previous work, where its content validity was established (Prasanna, Bajaj, et al., 2024). This information is referenced in the method section of the current study. Further evaluation of the tool's psychometric properties, specifically in terms of test-retest and inter-rater reliability, was conducted in the present research through a pilot study. The findings are detailed in the results section. The content has been updated accordingly to enhance clarity.

**3e. If the story was designed in English, how did the authors make sure that the translated version was faithful to the original version? It would be helpful to compare the psychometric properties of the original version to the translated version.**

**Reply:   **We apologize for the lack of clarity in our write-up, which led to this confusion. The present research employed the original English version of the tool, without any translations. Our study explored reasoning development among preschoolers using a story-based cognitive-communication assessment tool developed in our previous work (Prasanna, Bajaj, et al., 2024). The content validity of this tool was established in that prior research. In the current study, reliability measures for the reasoning tasks were established through a pilot study before assessing the reasoning abilities of the participants. The method section has been revised to avoid any confusion.

**3f. Can the authors present the findings of test-retest reliability for the pilot test on 15 children?**

**Reply:**  The test-retest reliability of the tool was established through the pilot testing on 15 children and the findings are presented in the results section.

1. **Results: The results are well presented, along with visual illustrations.**

**Reply: **Thank you for the kind feedback.

1. **Discussion**

**5a. The authors make a case that the findings can be attributed to biological maturation. The authors need to discuss the specific neurophysiological substrates that are correlated with higher-order tasks such as the one used in the current study.**

**Reply:** Thank you for the kind suggestion. The neurophysiological correlates of reasoning abilities among preschoolers, discussed in the present work, have been updated in the discussion section.
